# Idiopathic Foveal Cavitation in a Pediatric Patient: Multimodal Imaging Findings Mimicking Early Macular Hole Formation

**DOI:** 10.3390/diagnostics16131976

**Published:** 2026-06-25

**Authors:** Bogumiła Wójcik-Niklewska, Zofia Oliwa, Karina Dzięcioł, Mikołaj Gołda, Adrian Smędowski

**Affiliations:** 1 Department of Pediatric Ophthalmology, Faculty of Medical Sciences in Katowice, Medical University of Silesia, 40-055 Katowice, Poland; asmedowski@sum.edu.pl; 2 Professor Kornel Gibiński University Hospital Center, Medical University of Silesia, 40-514 Katowice, Poland; 3 Students’ Scientific Society, Department of Ophthalmology, Faculty of Medical Sciences in Katowice, Medical University of Silesia, 40-752 Katowice, Poland; zofiaoliwa2002@gmail.com (Z.O.); karina.dzieciol@o2.pl (K.D.); mikolajgolda01@gmail.com (M.G.); 4 The Laboratory for Translational Research in Ophthalmology, Department of Ophthalmology, Faculty of Medical Sciences in Katowice, Medical University of Silesia, 40-752 Katowice, Poland; 5 GlaucoTech Co., 40-282 Katowice, Poland

**Keywords:** pediatric ophthalmology, macular hole, foveal defect, optical coherence tomography, multimodal imaging

## Abstract

Macular holes are uncommon in pediatric patients and are most often associated with ocular trauma. Idiopathic cases are rare and may present as subtle clinical findings and atypical imaging features. We report a case of a 13-year-old boy presenting with decreased visual acuity in the left eye. Best-corrected visual acuity was 1.0 in the right eye and 0.5 in the left eye, with unremarkable anterior segment examination. Optical coherence tomography showed a foveal defect characterized by a central hyporeflective cavity with disruption of retinal layers, without evidence of a full-thickness defect. Fluorescein angiography demonstrated central hyperfluorescence without leakage. Color fundus photography revealed a subtle central foveal lesion, while electrophysiological testing and visual field examination were within normal limits. This case highlights that early structural abnormalities of the fovea in pediatric patients may present with minimal clinical findings and preserved retinal function. Multimodal imaging, particularly OCT, plays a key role in detecting subtle foveal alterations and may aid in identifying early stages within the spectrum of macular hole formation. Careful monitoring is warranted due to the potential for progression.

**Figure 1:**
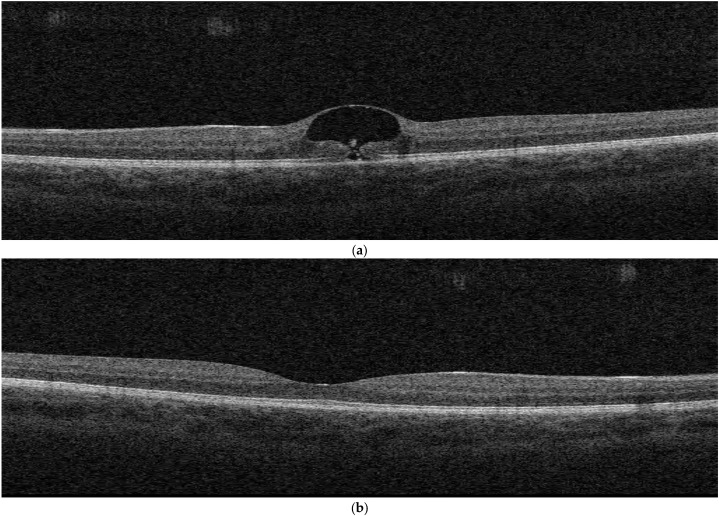
A 13-year-old boy presented for ophthalmological evaluation due to decreased visual acuity in the left eye. The patient’s medical history was unremarkable, with no previous history of ocular or head trauma, confirming the idiopathic nature of the presentation. Upon initial presentation, the uncorrected visual acuity (UCVA) was 5/5 in the right eye (OD) and 5/8 in the left eye (OS). Best-corrected visual acuity was 1.0 in the right eye with a refractive status of −0.25 DCyl ax 120° and 0.5 in the left eye with a refractive status of −0.75 DCyl ax 20°, indicating a mild myopic astigmatism rather than significant axial myopia. Anterior segment examination was unremarkable in both eyes. Intraocular pressure measured by applanation tonometry was within normal limits, recorded at 15 mmHg OD and 16 mmHg OS. Optical coherence tomography (OCT) of the left eye (**a**) revealed a foveal defect characterized by a central hyporeflective space with interruption of the neurosensory retinal layers. The lesion demonstrated well-defined margins with mild elevation of the surrounding retinal tissue. Disruption of the outer retinal layers, including the ellipsoid zone, was observed at the foveal center. These findings indicate early structural alteration of the fovea and may correspond to an early stage of macular hole formation [1,2]. OCT of the right eye (**b**) showed a normal foveal contour with preserved retinal architecture. Taken together, these multimodal imaging findings indicate localized structural alteration of the fovea with minimal clinical manifestation. Spectral-domain optical coherence tomography (SD-OCT) of the left eye revealed a foveal defect characterized by a central hyporeflective cavity with disruption of the neurosensory retinal layers (Figure 1). The lesion demonstrated features consistent with intraretinal structural alteration, including involvement of the outer retinal layers and disruption of the ellipsoid zone. No definite full-thickness retinal defect was identified. The retinal architecture outside the foveal region appeared relatively preserved. The fellow eye demonstrated normal foveal morphology. Fluorescein angiography of the affected eye demonstrated a subtle central hyperfluorescence without evidence of leakage (Figure 2). The surrounding retinal vasculature appeared unremarkable. These findings were non-specific and should be interpreted in correlation with OCT imaging. Color fundus examination showed a subtle central foveal lesion, while the fellow (right) eye was unremarkable with a preserved foveal reflex (Figure 3). Electrophysiological testing, including full-field electroretinography (ERG) and photopic negative response (PhNR), was within normal limits. Visual field testing did not reveal clinically significant defects, supporting the localized nature of the lesion and the preserved global retinal function. Regarding patient management, a conservative approach with close clinical observation was adopted. No immediate surgical or medical intervention was performed, and the patient was scheduled for regular follow-up evaluations to monitor for any structural progression or spontaneous resolution.

**Figure 2:**
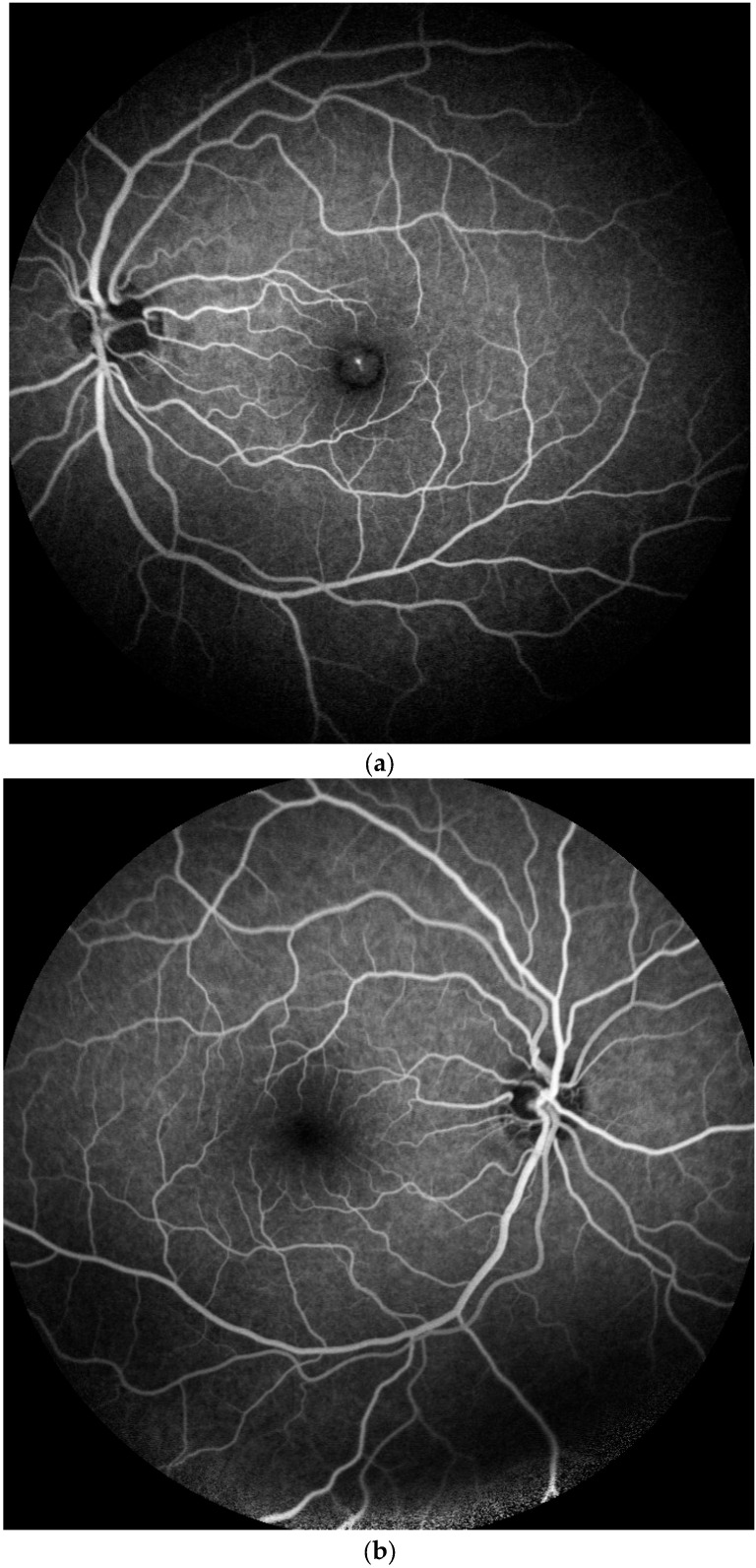
Fluorescein angiography of the left eye (**a**) demonstrated a central area of hyperfluorescence at the fovea, consistent with a window defect. The hyperfluorescence appeared stable without evidence of leakage throughout the angiographic phases. The surrounding retinal vasculature showed a normal pattern, with no signs of neovascularization. Fluorescein angiography of the right eye (**b**) was unremarkable.

**Figure 3:**
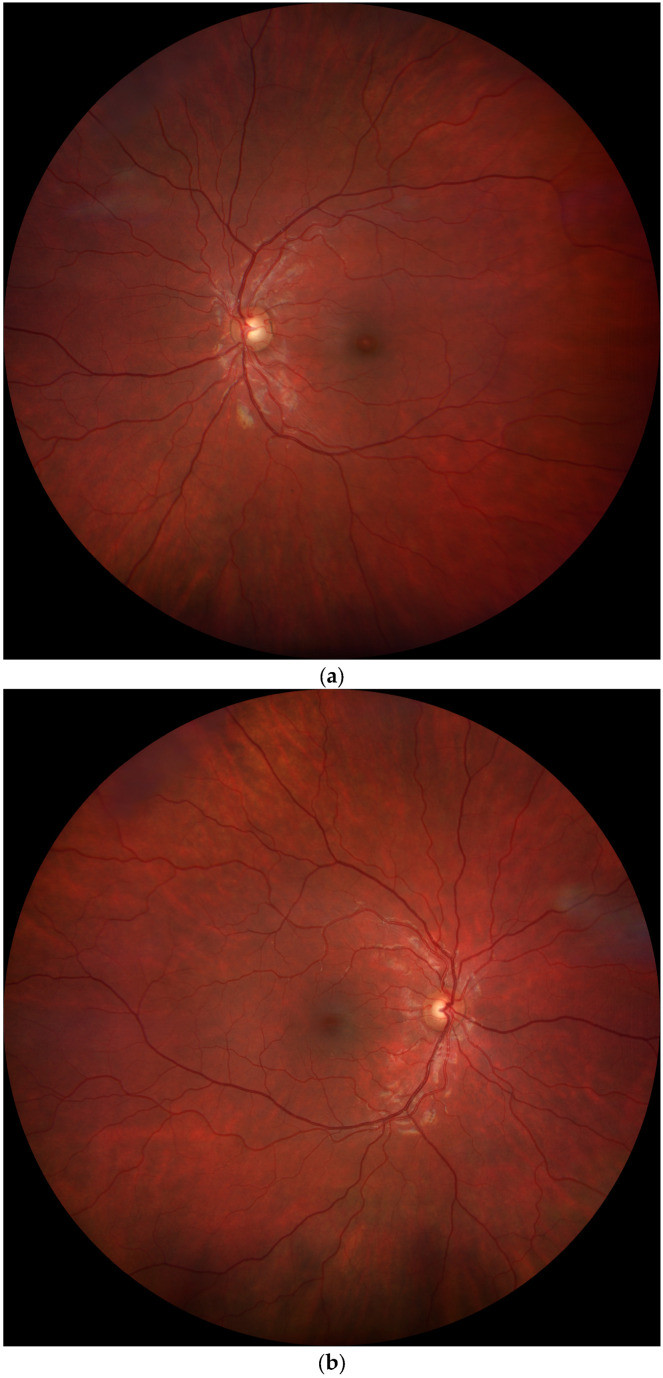
Color fundus of the left eye (**a**) demonstrated a well-circumscribed lesion at the foveal center, appearing as a small reddish to yellowish spot. The optic disk and retinal vessels were within normal limits. Color fundus photography of the right eye (**b**) was unremarkable. Macular structural abnormalities in pediatric patients are rare and differ from adult presentations. While macular holes in children are most commonly associated with trauma, idiopathic cases remain uncommon and may present with atypical imaging features [3,4,5,6,7]. The OCT findings in this case may represent an early stage within the spectrum of macular hole formation, reflecting structural instability at the vitreomacular interface [1,2,8]. However, the pathophysiology in pediatric patients remains incompletely understood. Differential diagnosis includes vitelliform lesions, which may present with subtle clinical findings and central macular changes. However, in contrast to vitelliform lesions, which typically demonstrate subretinal hyperreflective material, the present case showed intraretinal cavitation with disruption of retinal layers [9,10,11]. Juvenile retinoschisis was also carefully considered; however, it was ruled out due to the absence of characteristic wheel-spoke foveal schisis cavities in the inner nuclear or outer plexiform layers on OCT, normal electrophysiological findings, and the lack of systemic or family history suggestive of X-linked retinoschisis. Central serous chorioretinopathy is unlikely given the absence of leakage on fluorescein angiography and the patient’s age [12]. Another entity to consider is idiopathic internal limiting membrane (ILM) detachment. Li et al. described a rare case of idiopathic ILM detachment presenting with foveal elevation and OCT features resembling early macular hole formation without a full-thickness defect [13]. Although reported in adult patients, these findings may provide a useful reference for interpreting atypical foveal structural abnormalities in pediatric cases. The findings in the present case demonstrate a similar pattern of structural alteration in a younger patient. This case demonstrates that significant structural abnormalities of the fovea may be present in pediatric patients despite minimal clinical findings and preserved retinal function. The discrepancy between OCT and fundus appearance may lead to underdiagnosis if multimodal imaging is not performed. This case highlights the importance of OCT as a primary diagnostic tool in evaluating subtle macular abnormalities in children. It also expands the spectrum of idiopathic vitreomacular interface disorders in pediatric patients and emphasizes the need for careful monitoring due to the potential risk of progression. A limitation of this report is the absence of long-term follow-up imaging, which would allow assessment of lesion progression or potential spontaneous resolution. Additionally, OCT angiography was not performed and could have provided further insight into microvascular alterations. Genetic testing was not available; however, the imaging findings were not typical of inherited retinal dystrophies. Despite these limitations, multimodal imaging allowed accurate characterization of this rare condition.

## Data Availability

No new data were created or analyzed in this study. Data sharing is not applicable to this article.
